# Pentoxifylline and the proteasome inhibitor MG132 induce apoptosis in human leukemia U937 cells through a decrease in the expression of Bcl-2 and Bcl-XL and phosphorylation of p65

**DOI:** 10.1186/1423-0127-20-13

**Published:** 2013-02-28

**Authors:** Alejandro Bravo-Cuellar, Georgina Hernández-Flores, José Manuel Lerma-Díaz, Jorge Ramiro Domínguez-Rodríguez, Luis F Jave-Suárez, Ruth De Célis-Carrillo, Adriana Aguilar-Lemarroy, Paulina Gómez-Lomeli, Pablo Cesar Ortiz-Lazareno

**Affiliations:** 1 División de Inmunología, Centro de Investigación Biomédica de Occidente (CIBO), Instituto Mexicano del Seguro Social (IMSS), Sierra Mojada 800, Col. Independencia, Guadalajara, Jalisco 44340, México; 2 Departamento Ciencias de la Salud, Centro Universitario de los Altos, Universidad de Guadalajara, Tepatitlán de Morelos, Jalisco, México; 3 Departamento de Farmacobiología, Centro Universitario de Ciencias Exactas e Ingeniería, Universidad de Guadalajara, Guadalajara, Jalisco, México; 4 Programa de Doctorado en Ciencias Biomédicas Orientación Inmunología, Centro Universitario de Ciencias de la Salud, Universidad de Guadalajara, Guadalajara, Jalisco, 44340, México

**Keywords:** U937, Apoptosis-related genes, Caspases, p65 phosphorylation, Bcl-2, Bcl-XL, pentoxifylline, MG132

## Abstract

**Background:**

In Oncology, the resistance of the cancerous cells to chemotherapy continues to be the principal limitation. The nuclear factor-kappa B (NF-κB) transcription factor plays an important role in tumor escape and resistance to chemotherapy and this factor regulates several pathways that promote tumor survival including some antiapoptotic proteins such as Bcl-2 and Bcl-XL. In this study, we investigated, in U937 human leukemia cells, the effects of PTX and the MG132 proteasome inhibitor, drugs that can disrupt the NF-κB pathway. For this, we evaluated viability, apoptosis, cell cycle, caspases-3, -8, -9, cytochrome *c* release, mitochondrial membrane potential loss, p65 phosphorylation, and the modification in the expression of pro- and antiapoptotic genes, and the Bcl-2 and Bcl-XL antiapoptotic proteins.

**Results:**

The two drugs affect the viability of the leukemia cells in a time-dependent manner. The greatest percentage of apoptosis was obtained with a combination of the drugs; likewise, PTX and MG132 induce G1 phase cell cycle arrest and cleavage of caspases -3,-8, -9 and cytochrome *c* release and mitochondrial membrane potential loss in U937 human leukemia cells. In these cells, PTX and the MG132 proteasome inhibitor decrease p65 (NF-κB subunit) phosphorylation and the antiapoptotic proteins Bcl-2 and Bcl-XL. We also observed, with a combination of these drugs overexpression of a group of the proapoptotic genes *BAX*, *DIABLO*, and *FAS* while the genes *BCL-XL*, *MCL-1*, *survivin*, *IκB*, and *P65* were downregulated.

**Conclusions:**

The two drugs used induce apoptosis *per se*, this cytotoxicity was greater with combination of both drugs. These observations are related with the caspases -9, -3 cleavage and G1 phase cell cycle arrest, and a decrease in p65 phosphorylation and Bcl-2 and Bcl-XL proteins. As well as this combination of drugs promotes the upregulation of the proapoptotic genes and downregulation of antiapoptotic genes. These observations strongly confirm antileukemic potential.

## Background

Leukemia is a heterogenic group of diseases characterized by infiltration of neoplastic cells of the hematopoietic system into the blood, bone marrow, and other tissues [1,2]. Leukemia is the most common malignancy among people aged <20 years. In the last decade, these diseases have exhibited a clear ascending pattern in the morbidity index, becoming a great challenge to health institutions [3].

The main treatment for this disease is chemotherapy. However, its results are very often limited due to the treatment resistance that the neoplastic cells develop [4,5]. In an attempt to increase the efficiency of antileukemic treatments, higher doses of the cytotoxic agents have been used or different combinations of them [6,7], but in the majority of the cases, higher doses have been put into effect in an empirical manner without good results and incrementing side effects.

Given this situation, our research team has developed the concept of chemotherapy with a rational molecular basis. The former is based on the premise that chemotherapy acts mainly to induce a genetically programmed death of the cell called apoptosis, and that this depends in turn on the synthesis of proteins *de novo* and the activation of biochemical factors as a result of a modification in the balance between expression of pro- and antiapoptotic genes in response to treatment [8,9]. The cells undergoing apoptosis show internucleosomal fragmentation of the DNA, followed by nuclear and cellular morphologic alterations, which leads to a loss of the integrity of the membrane and the formation of apoptotic bodies. All of these processes are mediated by caspases, which are the main enzymes that act as apoptosis initiators and effectors. Some of these molecules can active themselves, while others require other caspases in order to acquire biological activity. This proteolytic cascade breaks down specific intracellular proteins including nuclear proteins of the cytoskeleton, endoplasmic reticulum, and cytosol, finally hydrolyzing the DNA [10-12].

On the other hand, it is noteworthy that upon apoptotic stimulus such as that generated by chemotherapy, this not only induces apoptosis but can also activate antiapoptotic mechanisms [13,14]. Similarly, the nuclear factor-kappa B (NF-κB) transcription factor plays an important role in tumor cell growth, proliferation, invasion, and survival. In inactive cells, this factor is linked with its specific inhibitor I-kappa B (IκB), which sequesters NF-κB in the cytoplasm and prevents activation of target genes [15-18]. In this respect, NF-κB can activate antiapoptotic genes such as *Bcl-2*, *Bcl-XL*, and *survivin*, affecting chemotherapy efficiency, even if the chemotherapy itself or the radiotherapy itself can activate the NF-κB factor [19-21]. Blast cells exhibit overexpression of antiapoptotic proteins (Bcl-2 and Bcl-XL), which increase resistance to antitumor therapy [22].

In this regard, the drug PTX can prevent the phosphorylation of serines 32 and 36 of IκB, and we have found that PTX in combination with antitumor drugs such as adriamycin and cisplatin induced *in vitro* and *in vivo* a significant increment of apoptosis in fresh leukemic human cells [8], lymphoma murine models [9], and cervical cancer cells [23]. Similar results have also been observed with PTX in other studies [24]. PTX is a xanthine and a competitive nonselective phosphodiesterase inhibitor that inhibits tumor necrosis factor (TNF) and leukotriene synthesis and reduces inflammation [25,26]. The MG132 proteasome inhibitor is another drug that decreases NF-κB activity [27]. Proteasome inhibitors are becoming possible therapeutic agents for a variety of human tumor types that are refractory to available chemotherapy and radiotherapy modalities [28,29]. The proteasome is a multicatalytic complex that is responsible for regulating apoptosis, cell cycle, cell proliferation, and other physiological processes by regulating the levels of important signaling proteins such as NF-κB, IκB, and the MG132 proteasome inhibitor have been shown to induce apoptosis in tumor cells [30,31]. This is important because apoptosis is regulated by the ubiquitin/proteasome system at various levels [32]. The aim of the present work was to study *in vitro* in U937 leukemic cells the effects on viability, apoptosis, cell cycle, caspases cleavage, cytochrome *c* release and mitochondrial membrane potential (ΔΨm), the Bcl-2 and Bcl-XL antiapoptotic proteins, and related genes activated by the PTX and/ or MG132 proteasome inhibitor, compounds that possess a NF-κB-mediated inhibitory effect.

## Methods

### Cells

The cell line U937 (ATCC CRL-1593.2), human monocytic leukemia, was used. These cells were cultivated in an RPMI-1640 culture medium (GIBCO, Invitrogen Co., Carlsbad, CA, USA) with the addition of 10% fetal bovine serum (FBS) (GIBCO), a 1% solution of L-glutamine 100X (GIBCO), and antibiotics (GIBCO), which will be designated as RPMI-S. The cells were maintained at 37°C in a humid atmosphere containing 5% CO_2_ and 95% air.

### Drugs

PTX (Sigma-Aldrich, St. Louis, MO, USA) was dissolved in a sterile saline solution (0.15 M) at a 200 mM concentration and stored at ‒4°C during a maximum period of 1 week. The MG132 proteasome inhibitor (N-CBZ-LEU-LEU-AL, Sigma-Aldrich) 0.5 mg was dissolved in 0.250 mL of Dimethyl sulfoxide (DMSO, Sigma-Aldrich), divided into 20 μL aliquots, and stored at ‒20°C. Immediately prior to use, this was diluted in RPMI-1640 culture medium at a final concentration of 1 μM.

### Cell culture and experimental conditions

U937 cells (2.5 × 10^5^-mL in T75 flasks, Corning Incorporated, Corning, NY, USA) were grown in RPMI-S for 24 hours and collected by centrifugation. The cells were reseeded onto 24 well plates; U937 cells were either treated with PTX (8 mM) or MG132 (1 μΜ), or PTX + MG132 (final concentrations). The cells were incubated with PTX for 1 hour prior to the addition of MG132. All experiments were carried out 24 hours after treatment, to exception of the p65 phosphorylation that it was analyzed 1 hour after treatment with PTX or MG132 and in the gene expression studies the cells were incubated with the drugs for only 3 hours. The concentrations of the treatments employed in this study were previously confirmed as being the most favorable for the induction of apoptosis in this experimental model [33,34].

### Cellular viability

Cell viability was determined at different times in U937 cells (2 X 10^4^). They were incubated with PTX, MG132 or PTX + MG132 during 18, 24, 36 and 48 hours, we use a WST-1 cell proliferation reagent commercial kit (BioVision, Inc. Milpitas, CA, USA) following the manufacturer’s instructions. This study is based on the reduction of tetrazolium salts (WST-1) to formazan. After of the incubation 10 μL/well of WST-1/ECS reagent was added and the U937 cell were incubated for another 3 hours. The absorbance was measured in a microplate reader (Synergy™ HT Multi-Mode Microplate Reader; Biotek, Winooski, VT, USA) at 450 nm as reading reference wavelength at 690 nm. Data are reported as the mean ± standard deviation of the optical density values obtained in each group.

### Cell cycle analysis by flow cytometry

For cell cycle analysis, the U937 cells were synchronized [35]. In brief, cells were culture in RPMI-1640 containing 5% FBS by 12 hours then the cells were washed and culture in RPMI-1640 containing 1% FBS overnight. After the cells were washed with PBS and changed to serum free medium for 18 hours, and finally the cells were passage and released into cell cycle by addition of 10% FBS in RPMI-1640 culture medium and 1 × 10^6^ cells were treated 24 hours with the different drugs. The BD Cycletest™ Plus DNA Reagent Kit was used following the manufacturer’s instructions (BD Biosciences, San Jose, CA, USA). DNA QC Particles (BD Biosciences) were used for verification of instrument performance and quality control of BD FACSAria I (BD Biosciences) cell sorter employed in DNA analysis. For each sample, at least 20,000 events were acquired and data were processed with Flowjo v7.6.5 software (Tree Star Inc., OR, USA).

### Assessment of apoptosis induction by PTX and MG132 proteasome inhibitor

Apoptosis was evaluated by means of the Annexin V-FITC FLUOS Staining kit (Annexin-V-Fluos; Roche, Mannheim, Germany). Briefly, 1×10^6^ U937 cells were treated 24 hours with PTX, MG132 or PTX + MG132 after that the samples were washed twice with PBS and resuspended in 100 μL of incubation buffer; 2 μL of Annexin V- Fluorescein Isothiocyanate (FITC) and 2 μL of propidium iodide (PI) solution were added. The samples were mixed gently and incubated for 10 min at 20°C in the dark. Finally, 400 μL of incubation buffer was added to each suspension, which was analyzed by flow cytometry. Annexin V- FITC-negative and PI-negative cells were considered live cells. Percentage of cells positive for Annexin V-FITC but negative for PI was considered to be in early apoptosis. Cells positive for both Annexin V-FITC and PI were considered to be undergoing late apoptosis and cells positive to PI were considered to be in necrosis. At least 20,000 events were acquired with the FACSAria I cell sorter and analysis was performed using FACSDiva software (BD Bioscience).

### Assessment of mitochondrial membrane potential by flow cytometry

U937 cells (1 × 10^6^) were treated 24 hours with the different drugs after that the cells were washed twice with PBS, resuspended in 500 μL of PBS containing 20 nM of 3,3-dihexyloxacarbocyanine iodide (DIOC_6_, Sigma-Aldrich), and incubated at 37°C for 15 min and the percentage of cells with ΔΨm loss was analyzed by flow cytometry. As an internal control of the disrupted ΔΨm, cells were treated for 4 hours with 150 μM of protonophore carbonyl cyanide m-chlorophenylhydrazone (CCCP, Sigma-Adrich) positive control. Flow cytometry was performed using FACSAria I (BD Biosciences). At least 20,000 events were analyzed with the FACSDiva Software (BD Biosciences) in each sample.

### Protein extraction for caspases-3, -8 and -9 and cytochrome *c* and Western blot assay

U937 cells (5 × 10^6^) were treated with PTX, MG132 and PTX + MG132 for 24 hours. After treatment, cells were harvested, washed twice with PBS and lysed with RIPA buffer (0.5% deoxycholate, 0.5% NP-40, 0.5% SDS, 50 mM Tris pH 7.4 and 100 mM NaCl) containing protein inhibitors. Following sonication (15 pulses, 50% amp), protein extracts were obtained after 30 min incubation at 4°C and 5 min of centrifugation at 14,000 rpm/4°C. Protein concentrations were determined using Dc Protein Kit (Bio-Rad Laboratories, Inc*.,* CA*,* USA). Total cell protein (40 μg) was subjected to electrophoresis using a 10% sodium dodecyl sulfate (SDS) polyacrylamide gel. Subsequently, proteins were transferred to Immobilon-P PVDF membranes (Millipore, Bedford, MA, USA) and incubated with 1× Western blocking reagent (Roche) during 1.5 hour for nonspecific binding. Immunodetection of caspases-3, -8 and -9 were performed using anti-caspases -3, -8 and -9 antibodies (BioVision, Inc.) and cytochrome *c* was effected using anti-cytochrome *c* antibody (Biolegend, San Diego, CA, USA) at 4°C overnight. After incubation with a horseradish peroxidase-conjugated secondary antibody (Santa Cruz Biotechnology, CA, USA) immunoreactive proteins were visualized by Western blotting luminol reagent using the ChemiDoc™ XRS equipment (Bio-Rad) with the Quantity One^®^ 1-d Analysis Software (Bio-Rad). Control β-actin antibody (Santa Cruz Biotechnology). Protein levels on Western blot were quantified using the IMAGEJ 1.46r package (NIH, Bethesda, MD, USA).

### Detection of Bcl-2 and Bcl-XL antiapoptotic proteins, and p65 phosphorylation by flow cytometry

For determination of Bcl-2, Bcl-XL, and phosphorylated p65, 1 × 10^6^ U937 cells were treated or not treated for 1 hour with PTX, MG132 or PTX + MG132. We employed Alexa Fluor^®^ 647mouse anti-human Bcl-2 and Alexa Fluor^®^ 647 mouse anti human Bcl-XL proteins (Santa Cruz Biotechnology) and Alexa Fluor^®^ 647 mouse anti-human NF-κB p65 (pS529) (BD Biosciences) antibodies. The staining procedures were according to protocol for detecting protein or activation of the phosphorylation state by flow cytometry. An appropriate isotype control was utilized in each test to adjust for background fluorescence, and the results are represented as the mean fluorescence intensity (MFI) of Bcl-2, Bcl-XL proteins, and phosphorylated p65 protein. For each sample, at least 20,000 events were acquired in a FACSAria I cell sorter (BD Biosciences) and data were processed with FACSDiva software (BD Biosciences).

### Quantitative real-time PCR

Total RNA of the U937 cells (5×10^6^) was obtained after 3 hours of incubation with the different treatments using the Purelink™ Micro-to-Midi purification system for total RNA (Invitrogen Co.). The DNAc was synthesized beginning with 5 μg of total RNA utilizing the Superscript™ III First-Strand Synthesis Supermix kit (Invitrogen Co.). Real-Time PCR was carried out with the System Light Cycler^®^ 2.0 (Roche Applied Science, Mannheim, Germany), for which we employed DNA Master plus SYBR Green I (Roche Applied Science). The PCR program consisted of an initial 10-min step at 95°C, and 40 cycles of 15-sec at 95°C, 5-sec  at 60°C, and 15-sec cycles at 72°C. Analysis of the PCR products was carried out with Light Cycler^®^ software (Roche Applied Science). Data are presented in relative normalized quantities employing *L32* ribosomal gene expression to verify the specificity of the amplified reaction, which was nearly 100%. The oligonucleotides (Invitrogen Co.) were designed in the data base of nucleotides of the Gen Bank of the National Information Center for Biotechnology (http://www.ncbi.nlm.nih.gov) using the oligo v.6 program (Table 1).

**Table 1:** Primer pair used for real-time (RT) quantitative PCR

**Gene**	**Primer pair sequences**	**Gen bank accession No.**
*BAK*	5^′^CGC TTC GTG GTC GAC TTC AT 3^′^	NM001188
	5^′^AGA AGG CAA AGA CTT CGC TTA 3^′^	
*BAX*	5^′^TTT GCT TCA GGG TTT CAT CC 3^′^	NM138764
	5^′^CAG TTG AAG TTG CCG TCA GA 3^′^	
*DIABLO*	5^′^TGA CTT CAA AAC ACC AAG AGT A3^′^	NM019887
	5^′^TTT CTG ACG GAG CTC TTC TA 3^′^	
*DR4*	5^′^CTC GCT GTC CAC TTT CGT CTCT3^′^	NM003844
	5^′^GTC AAA GGG CAC GAT GTT3^′^	
*FAS*	5^′^TGA ACA TGG AAT CAT CAA GGA3^′^	NM000043
	5^′^CAA AGC CTT TAA CTT GAC TT3^′^	
*BCL-XL*	5^′^GCA GGC GAC GAG TTT GAA CT 3^′^	NM138578
	5^′^GTG TCT GGT CAT TTC CGA CTG A 3^′^	
*MCL-1*	5^′^CAC GAG ACG GTC TTC CAA GGA TGC T 3^′^	NM021960
	5^′^CTA GGT TGC TAG GGT GCA ACT CTA GGA 3^′^	
*SURVIVIN*	5^′^TGA GCT GCA GGT TCC TTA TCT G 3^′^	NM001168
	5^′^GAA TGG CTT TGT GCT TAG TTT T 3^′^	
*IkBa*	5^′^GGA TAC CTG GAG GAT CAG ATT A 3^′^	NM001278
	5^′^CCA CCT TAG GGA GTA GTA GAT CAA T 3^′^	
*P65*	5^′^GCA GGC TCC TGT GCG TGT CT 3^′^	NM02975
	5^′^GGT GCT CAG GGA TGA CGT AAA G 3^′^	
*RPL32*	5^′^GCA TTG ACA ACA GGG TTC GTA G 3^′^	NM000994
	5^′^ATT TAA ACA GAA AAC GTG CAC A 3^′^	

Oligonucleotides were designed using the Oligo v.6 software. Gene sequences were obtained from the GenBank Nucleotide Database of the National Center for Biotechnology Information (NCBI) (http://www.ncbi.nlm.nih.gov).

### Statistical analysis

All experiments were carried out in triplicate and were repeated three times. The values represent mean ± standard deviation of the values obtained. Statistical analysis was performed with the non-parametric Mann-Whitney *U* test considering *p* <0.05 as significant. In some experiments, we calculated the ∆%, which represents the percentage of increase or diminution in relation to the corresponding untreated control group (UCG). For the different gene expressions, we considered significant variations as ≥ at 30% compared with the constitutive gene [8].

The committee of ethics, biosafety and research of CIBO approved the study with the number 1305-2005-16.

## Results

### PTX and MG132 proteasome inhibitor induce a decrease in viability in U937 cells

**Figure 1:**
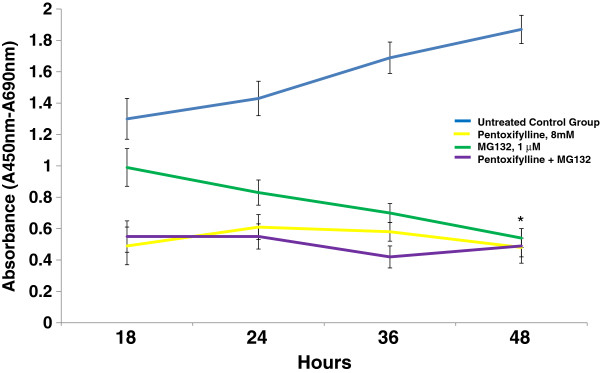
**Evaluation of viability in U937 human leukemia cells treated with PTX, MG132 and PTX + MG132. **U937 cells were incubated in the presence of PTX, MG132, or PTX + MG132 for 18, 24, 36, and 48 hours as previously indicated. After incubation, WST-1 was added and 3 hours later, viability was assessed by spectrophotometry at 450 nm. The results represent mean ± standard deviation of three independent experiments performed in triplicate. Statistical analysis Mann-Whitney *U *test. *♦ p *<0.05 PTX or PTX + MG132 *vs *untreated control group or MG132 group. ● *p *<0.05 PTX + MG132 *vs *all groups. * *p* < 0.05 PTX, MG132 or PTX + MG132 *vs *untreated control group.

We evaluated the effect on viability of U937 leukemic cells treated with both drugs. PTX, MG132, or PTX + MG132 induce inhibition of cell viability in time-dependent manner (Figure 1). In the case of PTX or PTX + MG132 treated cells, these treatments at 18 hours exhibited similar behavior inducing around 60% of diminution of cell viability (*p* < 0.05 *vs* all groups). These values practically did not change in the other times. In contrast, at this same time the cellular viability was slightly modified by MG132 treatment (*p* <0.05 *vs* other treated groups) and reached similar values to those of the other two treated groups at 48 hours after treatment (optical density = PTX 0.48 ± 0.06, MG132 0.54 ± 0.06, PTX + MG132 0.49 ± 0.11, *p* < 0.05 vs untreated control group 1.87 ± 0.9).

### PTX and MG132 proteasome inhibitor induce G1 cell cycle arrest in U937 cells

**Figure 2:**
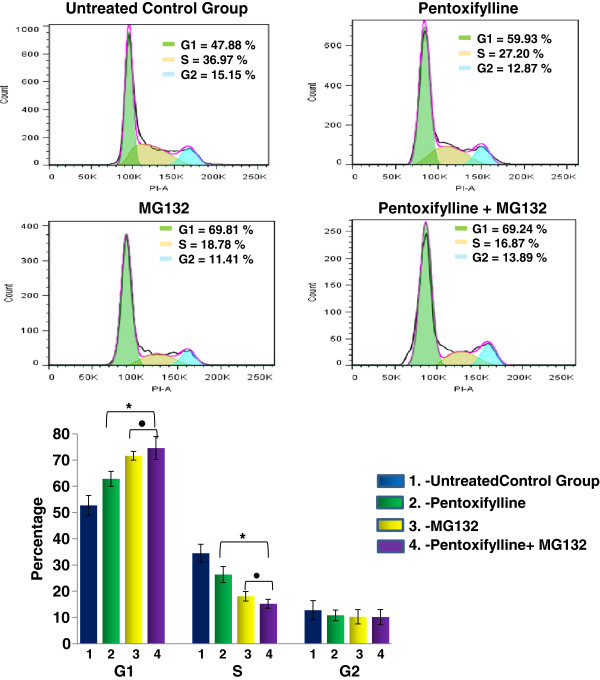
**PTX and MG132 modulate the cell cycle in U937 cells. **U937 cells were incubated alone or were treated with 8 mM PTX, 1 μM MG132, or PTX + MG132 for 24 hours as previously indicated. After incubation the cell cycle was analyzed by flow cytometry. The results represent mean ± standard deviation of three independent experiments performed in triplicate. Statistical analysis Mann-Whitney *U *test * p <0.05 PTX, MG132 or PTX + MG132 *vs *untreated control group. ● *p *<0.05 MG132 or PTX + MG132 groups *vs *PTX or untreated control group.

Our next interest was to elucidate whether the combination PTX + MG132 modulates the cell cycle. To address this point, U937 cells were treated in similar conditions with PTX, MG132 or PTX + MG132 for 24 hours and, subsequently, flow cytometry analysis of DNA content to determine cell populations in the different cell cycle phases was performed. As depicted in Figure 2, the percentage of untreated control group in G1 phase was 52.7 ± 3.8%. This percentage of cells is increased in PTX treated group Δ% = 25% and the maximum increment was observed in MG132 and PTX + MG132 treated groups with nearly to Δ% = 45% for both groups *p* < 0.05. For the S phase opposite results were observed, and it was found 34.5 ± 3.4% of U937 tumor cells in phase S; however, the Δ% in PTX, MG132 or the combination of both drugs were - 26.4%, -49.2% and -54.3% respectively *p* < 0.05. Finally for the G2 phase the percentage of cells from untreated control group was 12.8 ± 3.6%, it diminished in treated groups Δ% = -15.2%, -24.5%, -10.9% for PTX, MG132 and PTX + MG132 groups respectively. These observations suggest that PTX and MG132 or its combination induce a cell arrest in the G1 phase.

### Apoptosis induction by PTX + MG132

**Figure 3:**
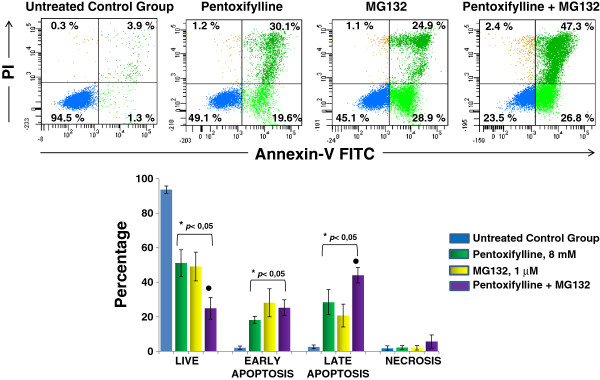
**Induction of apoptosis in U937 cells treated with PTX, MG132 and PTX + MG132. **U937 cells were incubated exclusively in RPMI-S culture medium or were treated with PTX, MG132, or PTX + MG132 for 24 hours. After incubation apoptosis was assessed using Annexin V-FITC/PI. The results represent mean ± standard deviation of three independent experiments performed in triplicate. Mann-Whitney *U *test. *p <0.05 all groups *vs* untreated control group; ●*p *<0.05 PTX + MG132 *vs* all groups.

At 24 hours of culture, apoptosis was evaluated in the U937 human leukemia cells that was induced by the different treatments under experimental conditions as previously described. In Figure 3, it is observed that the untreated control group showed a low percentage of early and late apoptosis (2.1 ± 0.9% and 2.6 ± 1.1% respectively) compared with the group treated exclusively with either PTX (18.2 ± 2.1% and 28.5 ± 7.3% of early and late apoptosis, respectively, *p* <0.05), or treated with MG132 proteasome inhibitor so we observed 28.1 ± 8.1% and 20.7 ± 6.6% of early and late apoptosis, respectively (*p* < 0.05 *vs* untreated control group). It was also very interesting to observe that the group of cultures exposed to PTX + MG132 showed a greater percentage of late apoptosis 44.1 ± 4.5% in comparison with all other groups *p* <0.05.

### PTX + MG132 induce mitochondrial membrane potential (ΔΨm) loss

**Figure 4:**
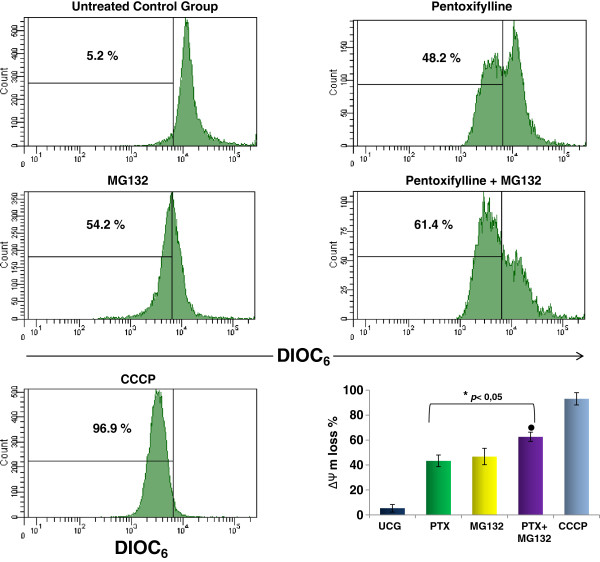
**PTX + MG132 induces loss of the mitochondrial membrane potential (ΔΨm). **U937 cells were cultured and treated with 8 mM PTX, 1 μM MG132, or PTX + MG132. After 24 hours, the cells were harvested and the ΔΨm was assessment by flow cytometry using DIOC_6_ staining. Protonophore carbonyl cyanide m-chlorophenylhydrazone (CCCP) was used as a positive control. The results represent mean ± standard deviation of three independent experiments performed in triplicate. Statistical analysis Mann-Whitney *U *test. * *p *<0.05 all groups *vs *untreated control group (UCG); ●  *p *<0.05 PTX + MG132 *vs *all groups.

As mitochondria plays an important role in apoptosis, for that reason we determined the ΔΨm in U937 leukemia cells treated with PTX, MG132 or PTX + MG132 and the results are represented in the Figure 4. The ΔΨm did not change in untreated control group. However when the cells were treated with either PTX or MG132 an important loss of the ΔΨm were noted 43.4 ± 4.7% and 46.8 ± 6.6 respectively (*p* < 0.05 compared with untreated control group), and it is interesting that PTX + MG132 induce an important ΔΨm loss in U937 cells 62.7 ± 3.7%, in comparison with the other groups *p* < 0.05.

### PTX + MG132 increase cleavage in caspases-3, -9 and cytochrome *c* release

**Figure 5:**
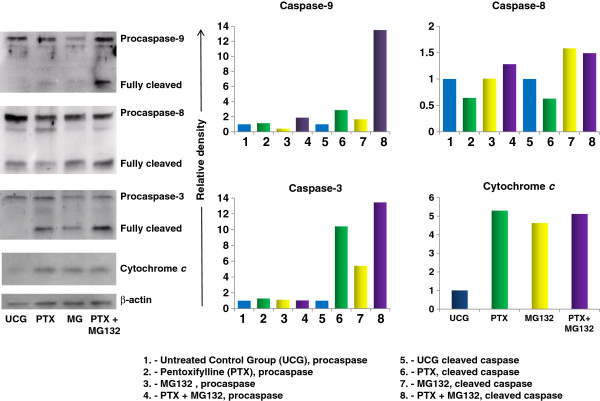
**Western blot analysis of caspases -3,-8,-9 and cytochrome *c *in U937 cells treated with PTX, MG132 or PTX + MG132. **U937 cells were cultured and treated with 8 mM PTX, 1 μM MG132, or PTX + MG132. After 24 hours, the cells were harvested and lysed. Equivalent amounts of individual lysates were placed on 10% SDS gradient polyacrylamide gels for electrophoresis and then were electrotransferred to Immobilon-P PVDF membranes. A representative study is shown and two additional experiments yielded similar results.

We determined caspases -3, -8, -9 and cytochrome *c* by Western blot. The analysis reveals that the combination PTX + MG132 was more effective in the activation of caspases-9 and -3. The results in Figure 5 allow us to observe that PTX increase cleavage of caspases-9 (2.8 fold) and -3 (10.4 fold), and the release of cytochrome *c* (5.2 fold) compared with untreated control group *p* < 0.05. In similar way MG132 proteasome inhibitor increase cleavage of caspase-3 in 5.4 fold, caspase-9 in 1.7 fold and caspase-8 in 1.4 fold change and release of cytochrome *c* in 4.8 fold compared with untreated control group *p* < 0.05. It is important to stress that when we used PTX + MG132 we observed considerably cleavage of caspase-9 (13.5 fold) and caspase-3 (13.4 fold) compared with PTX or MG132 alone and with untreated control group, *p* < 0.05. In the same way, when we use both drugs simultaneity we observed an increase in the release of cytochrome *c* (5.11 fold) and cleavage of caspase-8 (1.88 fold) in comparison with untreated control group *p* < 0.05.

### Determination by flow cytometry of phosphorylated p65 protein from NF-κB, Bcl-2 and Bcl-XL antiapoptotic proteins

**Figure 6:**
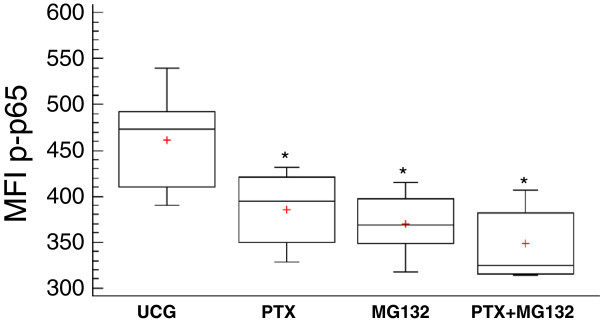
**Determination of phosphorylated p65 (NF-κB subunit) in U937 cells treated with PTX, MG132 and PTX + MG132. **U937 cells were incubated either alone or treated with 8 mM PTX, 1 μM MG132, or PTX + MG132. After 1 hour, the phosphorylated p65 protein was determined by flow cytometry. For each sample at, least 20,000 events were acquired. The results represent mean ± standard deviation of the Mean Fluorescence Intensity (MFI) of phosphorylated p65 of three independent experiments carried out in triplicate. *p <0.01 *vs *the untreated control group.

**Figure 7:**
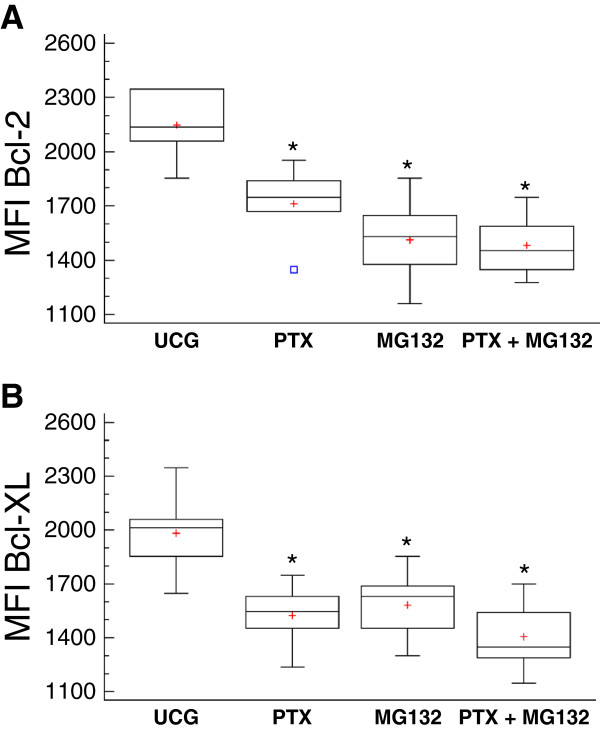
**PTX and MG132 reduce the expression of Bcl-2 and Bcl-XL antiapoptotic proteins in U937 cells. **U937 human leukemia cells were incubated alone or were treated with 8 mM PTX, 1 μM MG132, or PTX + MG132. After 24 hours the Bcl-2 (**A**) and Bcl-XL (**B**)antiapoptotic proteins were determined by flow cytometry. For each sample, at least 20,000 events were acquired. The results represent mean ± standard deviation of the Mean Fluorescence Intensity (MFI) of Bcl-2 or of Bcl-XL of three independent experiments carried out in triplicate. *p <0.01 *vs *the untreated control group.

The phosphorylated p65 protein was quantified determining the Mean Fluorescence Intensity by flow cytometry. As we expected, in comparison with the Untreated Control Group, Figure 6 shows that U937 human leukemia cells treated with PTX or the MG132 proteasome inhibitor decrease the phosphorylation of p65 (*p* <0.05), and in the combination of both compounds, this diminution is more pronounced. The antiapoptotic proteins Bcl-2 and Bcl-XL play a transcendent role in chemoresistance in tumor cells; therefore, these proteins could be regulated by the NF-κB transcription factor. For this, we studied the effect of PTX and MG132 in these proteins. We can observe in Figure 7A that tumor U937 cells treated with PTX, MG132, or PTX + MG132 in a similar manner reduce the expression of Bcl-2 protein in comparison with the untreated control group (*p* <0.05). In the same way, in Figure 7B, we can see that when U937 cells were treated with the same schedule of treatments. We also observed a reduction in Bcl-XL in comparison with the untreated control group (*p* <0.05), with a tendency to be the most pronounced in the group treated with both drugs. These results together are according with apoptosis, caspases cleavage, and cytochrome *c* release and ΔΨm loss experiments and strongly suggest that assayed treatments inhibited the expression of important proteins related with the survival of the cells, being most important with the combination of the drugs.

### Changes in the expression of proapoptotic, antiapoptotic, and NF-κB-related genes

**Figure 8:**
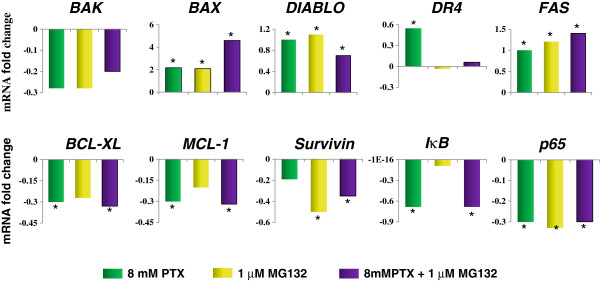
**Expression of pro- and antiapoptotic genes in U937 cells treated with PTX, MG132 and PTX + MG132. **U937 human leukemia cells were incubated alone or treated with 8 mM PTX, 1 μM MG132, or PTX + MG132. After 3 hours gene expression was assessed by quantitative Real-Time PCR. The data are expressed as messenger (mRNA) fold change in relative normalized quantities employing the RPL32 gene expression. In all cases, standard deviation was not >0.08. Arbitrary was considered as significant upregulation or downregulation when the change was ≥ 30% in relation to constitutive gene expression.

Real-Time PCR was employed to determine relative change in gene expression (Figure 8). Arbitrary was considered as significant upregulation or downregulation when the change was ≥ 30% in relation to constitutive gene. In PTX-treated U937 cells, we found upregulation of *BAX*, *DIABLO*, *DR4*, and *FAS* proapoptotic genes in comparison with untreated control group, and the most important upregulation observed with *BAX* (2.17-fold upregulation). Similarly, PTX induces downregulation of *BCL-XL* and *MCL-1* antiapoptotic genes and of *IκB* and *p65* NF-*κB*-related genes. When U937 culture cells were treated with the MG132 proteasome inhibitor, we observed upregulation of *BAX*, *DIABLO*, and *FAS* genes. In the case of antiapoptotic genes, MG132 induces downregulation of *Survivin* and *p65* genes. When the cell cultures were treated with PTX + MG132 we observed upregulation of the proapoptotic genes *BAX* with the greatest upregulation (4.6-fold upregulation), and with *FAS* and *DIABLO genes*. In relation to PTX + MG132-treated U937 culture cells antiapoptotic genes *BCL-XL*, *MCL-1*, and *Survivin* were downregulated as well as the NF-κB-related genes *IκB* and *p65*. In general, with these treatment schedules the data suggest a balance in favor of proapoptotic genes in U937 human leukemia cells treated with PTX + MG132.

## Discussion

In the present work, we studied the viability of U937 human leukemia cells treated with PTX and/or MG132 using the spectrophotometric assay of WST-1 as well as apoptosis by flow cytometry. These results are in agreement between them and with prior experiments clearly showing that PTX and MG132 possess an important antitumor activity *per se*, as has been reported [24,36]. This increasing in cytotoxicity when the drugs are added simultaneously to tumor cell cultures in an important manner, suggests an additive effect. In addition, the fact of having found a clear effect of time-dose dependence speaks to the specificity of the treatments. In this respect, the potential of PTX and MG132 is great because there reports of successful combinations of PTX with antitumoral drugs such as adriamycin [8] and cisplatin [23], and MG132 can synergize the antitumoral activity of TRAIL receptor agonist [37] and propyl gallate [38]. In these sense our study conincide with these reports because we observe an important induction of late apoptosis (44.1%) when we use the combination PTX + MG132 in U937 leukemia cells.

The growth arrest of tumor cells in G1 phase provides an opportunity for cells to either undergo apoptosis or induce cell repair mechanisms [39,40]. Interestingly, in our study we observed with the different treatment arrest in G1 phase and apoptosis induction. In this point apparently the lower percentages of cells in S phase are due to MG132 effect because the percentage of cells treated exclusively with the proteasome inhibitor shows the same values than the cells treated with PTX + MG132, suggesting different action mechanisms between two drugs.

Based in the correlation of our observations related with the ΔΨm loss, cytochrome *c* release, caspase assays we think that apoptosis observed it is due principally to the mitochondrial pathway. In addtion these results together are in aggremeent with previously reports [41,42].

It is known that PTX prevents the activation of NF-κB by avoiding the breakdown of its inhibitory molecule, IκB [43]; MG132 is also an NF-κB inhibitor as well as of the proteasome [44]. We used both drugs in our experiments in order to observe the modifications in p65 (NF-κB subunit) phosphorylation. In U937 leukemic cells, we found a decrease in p65 phosphorylation with PTX and MG132 or its combination compared with untreated cells (*p* < 0.05). The fact that the experimental treatment induces a decrease in NF-κB phosphorylation allows us to suppose the presence of important alterations in a mechanism that promotes resistance to antitumor therapy [45,46].

We decided to study the Bcl-2 and Bcl-XL proteins that possess antiapoptotic activity that can be regulated by NF-κB activation [47,48]. In others tumor cells have shown an overexpression of these proteins promoting a resistance to radiotherapy or chemotherapy [49,50]. Likewise, some studies have reported that various chemotherapeutic agents commonly used upregulated Bcl-2 and Bcl-XL expression through the NF-κB-dependent pathway [51,52]. These proteins suppress apoptosis by preventing the activation of the caspases that carry out the process [53,54]. The susceptibility in U937 leukemia cells to apoptosis induced by PTX and MG132, it can explain for the decrease in the expression of Bcl-2 and Bcl-XL proteins when the cells are expose to both drugs. Moreover the decrease in the levels of Bcl-2 leads to ΔΨm loss potential. This fact is key event for the apoptosis induction [55]. The data suggest that PTX + MG132 treatment induces caspases-dependent mitochondrial intrinsic pathway because we found disruption in mitochondrial membrane potential, cytochrome *c* release and an important cleavage of caspases-9 and it is well known that it leads to caspase –3 cleavage and apoptosis induction [56]. Our result show that the proapoptotic genes exhibited upregulation with the different treatments and this tendency is observed mainly in *BAX*, *DIABLO*, and *FAS* genes. Contrarily, the antiapoptotic genes were downregulated, mainly *BCL-XL*, *MCL-1*, and *survivin*.

It is important to stress that in relation to proapoptotic genes study we found the highest upregulation in the *BAX gene* and this is in agreement with our data in relationship to the mitochondrial pathway participation observed in this paper.

Above suggests that there is a gene balance that favors apoptosis induction. We found a downregulation in the *IκB* when leukemia cells were treated with PTX or PTX + MG132 and in *p65* genes when U937 leukemic cells were treated with PTX, MG132, or its combination, suggesting a diminution of the biological availability of these factors that facilitate cell death.

## Conclusion

Our results show that in this experimental model with U937 human leukemia cells, PTX and MG132 showed antileukemic activity, and together have an additive effect. These drugs disturb the NF-κB pathway and induce cell arrest in G1 phase, and decrease of antiapoptotic proteins Bcl-2 and Bcl-XL and induce ΔΨm loss, cytochrome c release and a caspases-3,-9,-8 cleavage resulting in an increase in apoptosis. In addition the different treatments gave rise to equilibrium in favor of the expression of proapoptotic genes. For these previously mentioned reasons, in general our results support the idea that chemotherapy must be administered under rational molecular bases.

## Competing interests

The author declares no potential conflict of interests.

## Authors’ contributions

PCO-L, AB-C, and GH-F designed and performed the research, analyzed the data, and drafted the manuscript; JML-D, JRD-R, PG-L, and RC-C performed some of the research and analyzed the data, and AA-L and LFJ-S conducted the molecular study and analyzed the data. All of the authors read and approved the final manuscript.
